# Craniofacial genetics: Where have we been and where are we going?

**DOI:** 10.1371/journal.pgen.1007438

**Published:** 2018-06-21

**Authors:** Seth M. Weinberg, Robert Cornell, Elizabeth J. Leslie

**Affiliations:** 1 Department of Oral Biology, University of Pittsburgh, Pittsburgh, Pennsylvania, United States of America; 2 Department of Human Genetics, University of Pittsburgh, Pittsburgh, Pennsylvania, United States of America; 3 Department of Anthropology, University of Pittsburgh, Pittsburgh, Pennsylvania, United Staes of America; 4 Department of Anatomy and Cell Biology, University of Iowa, Iowa City, Iowa, United States America; 5 Department of Human Genetics, Emory University, Atlanta, Georgia, United States of America

Looking at faces is always illuminating. Perhaps, this is because our faces reveal so much about us, ranging from our evolutionary history to our embryological development, genetic endowment, propensity for disease, current health status, and exposures over our lifespan. The structure of our faces may even reveal insights into our personalities—an idea that stretches back to the ancient Greeks. The face is a complex constellation of parts serving functions as diverse as sight, hearing, smell, breathing, nourishment and digestion, protection, and communication. Despite our collective fascination, we still have limited understanding of the molecular machinery that controls how our faces form or how morphological variation in facial features arises, from the typical and often subtle differences that endow each of us with our unique facial appearance to the rare craniofacial malformations seen in the clinic. However, we are making incredible progress in these areas, and the pace of discovery is poised to accelerate rapidly, facilitated by the emergence of high-throughput experimental methods, advances in computational modeling, and the investment and availability of large-scale craniofacial data resources, (e.g., the FaceBase Consortium).

In recognition of these advances, *PLOS Genetics* invited us to put together a special collection on the theme of craniofacial genetics. This collection was not meant to be exhaustive but rather a curated selection of published papers covered in PLOS journals deemed (by us) to be particularly germane. Thus, the selection is, by its nature, highly subjective, reflecting our particular areas of interest: craniofacial morphogenesis, dysmorphology and syndromes, and normal human facial variation.

## The genes and pathways controlling craniofacial morphogenesis: Zebrafish as a model

The zebrafish has emerged as a potent addition to the traditional mouse model for studies of craniofacial morphogenesis. The main features include external development and optical transparency, which both facilitate imaging of live embryos and amenability to forward and reverse genetic methods. Also, the zebrafish larval face is simpler than that of a newborn human at the time feeding begins [1]. Many of the mechanisms governing early growth and patterning of the face are shared across all vertebrates. Although there are unique structures in zebrafish, mice, and humans, fundamental cellular processes are conserved. For instance, convergence of frontonasal and maxillary neural crest occurs analogously in the formation of the mammalian hard palate and the zebrafish ethmoid plate. The soft palate, separating the nasal cavity and the oral cavity in mammals, is absent in zebrafish. Nonetheless, zebrafish may still be useful in studying genetic underpinnings of cleft (soft) palate, as the gene regulatory networks governing differentiation of relevant tissues are similar between zebrafish and mammals [2]. Exemplifying this point, the gene regulatory network governing differentiation of the mammalian superficial oral epithelium (oral periderm), where a large subset of genes associated with risk for cleft palate are expressed, has many shared features with that of zebrafish periderm [3–6]. This means that many insights into zebrafish development, gleaned from its impressive experimental toolkit, will be relevant to the development of the mammalian face and, in some cases, to the genetic underpinnings of orofacial clefting.

The external development and transparency of zebrafish embryos permits exquisite imaging by high-resolution confocal microscopy. One application is fate mapping using photo-convertible dyes [7]. Reporters built from the regulatory elements of appropriate genes have been used extensively to mark all neural crest cells [8,9], and, more recently, subsets of them that are differentiated as chondrocytes [10] or bone [11]. Use of such tools, and vital dyes, in normal animals has enabled the study of changes in cell organization that occur as a stream of cranial neural crest converts into a skeletal element of precise size and shape [11]. Similar analyses in mutants permit identification of the exact steps that go awry, facilitating identification of the underlying cellular defects [12]. Another potent application of imaging is the development of reporter lines that reveal a response to particular extracellular signals relevant to craniofacial patterning, including BMPs [13], FGFs [14], WNTs [15], and Hedgehog [16]. These tools permit investigations that would be much harder to carry out in mice.

Another experimental strength of zebrafish is that they are well suited to forward genetic screens, a potent method to identify genes involved in craniofacial development, including of the face. Even in an era where targeted mutagenesis is becoming increasingly efficient, random mutagenesis screens make an important contribution because they can yield alleles that would never be generated intentionally. Hypomorphic alleles can reveal a function in craniofacial morphogenesis for a gene product in which a total loss of function allele leads to death prior to craniofacial development. Morphology-based screens in the late 1990s revealed more than 80 mutants (as complementation groups) with grossly defective head shape [17–20]. Molecular characterization of mutants from these screens and subsequent ones that evaluated subtler phenotypes based on gene expression and histology revealed a large number of genes previously shown to be essential for development of the mammalian face, showing that these pathways are conserved. Excitingly, such efforts have also identified several genes not previously implicated in craniofacial development. Such genes include histone variant H3.3 [21], Rabconnectin-3a [22], and Hdac1 [23]. These genes are likely to be important for mammalian facial development as well. There is no reason to think such screens have been saturated, and additional, more elaborate reporter-based screens may reveal genes missed in earlier screens.

Zebrafish are also useful for loss-of-function studies; until recently, this was most frequently achieved by antisense morpholinos but increasingly by targeted mutagenesis mediated by clustered regularly interspersed short palindromic repeats (CRISPR)/Cas9 [24]. There is debate in the field regarding why the phenotypes induced by morpholinos and those induced by targeted mutagenesis are frequently not congruent, with the latter often less severe. One possibility is that the morphant phenotype is an artifact from an off target effect [25]; another is that targeted mutagenesis, but not morpholinos, induces a compensatory mechanism [26, 27]. Currently, a conservative approach is to use morpholinos only when they phenocopy a mutant [28]. More than 30 genes associated with craniofacial disorders also result in craniofacial dysmorphogenesis in zebrafish [1,29]. In many cases, confirmation of the morpholino oligonucleotide-induced phenotype in a targeted mutant remains to be achieved. Once it has been, the exquisite imaging methods available in zebrafish should permit relatively facile discovery of the function of each of these genes in craniofacial development.

## The genetics of craniofacial malformations and syndromes

Over the last 15 years, we have witnessed a dramatic increase in gene discovery for all manners of craniofacial phenotypes due to the development and application of high-throughput genomic technologies. Beginning in 2005, genome-wide association studies (GWAS) have produced strong evidence that common DNA sequence variants influence risk of common traits and diseases. The first application of GWAS to craniofacial phenotypes came in 2009 with back-to-back publications on nonsyndromic orofacial clefts (OFCs) [30,31]. Additional GWAS have been published at a consistent rate, collectively identifying over 25 risk loci across diverse population groups [32–43]. The more recent application of next-generation sequencing (NGS) technology welcomed rapid gene discovery for rare diseases, including those with extreme rarity, clinical heterogeneity, and those without strong family history that were not amenable to linkage. The major contribution of GWAS and discovery of etiological mutations by NGS has been the identification of novel candidate genes and new hypotheses about the mechanisms contributing to craniofacial development; these discoveries complement the insights gained from forward genetic screens in zebrafish mentioned above.

Novel findings for both common and rare craniofacial disorders have provided new etiological hypotheses and new insights into craniofacial phenotypes but also present new challenges for research. First, the discovery of new candidate genes has outpaced the functional studies required to understand the cellular and molecular mechanisms for both common and rare diseases. This is especially true for etiological variants occurring in noncoding regulatory parts of the genome, which we know considerably less about and for which we lack robust bioinformatic tools for annotation. Second, both GWAS and NGS have revealed a complexity to phenotypic presentations. For GWAS, identified loci may act broadly on the craniofacial complex, while others have very precise effects. In the example of OFCs, the *FOXE1* locus on 9q22 increases risk for all types of OFCs (including cleft palate only) [34, 44], whereas the *GREM1* locus on 15q13 appears to have specificity for cleft lip and palate [45]. For rare diseases, NGS has clarified unusual presentations of syndromes. In some cases, sequencing reveals multiple, independent molecular diagnoses in individuals previously thought to have a novel or new variation of a single syndrome. As a result, we foresee a growing list of genes in which genetic variants contribute to multiple diseases, including syndromic and nonsyndromic forms of craniofacial disorders, leading to new questions as to the mechanisms by which certain genotypes lead to observed phenotypes. Addressing these questions will require continued detailed phenotyping, large-scale genotyping and sequencing efforts, the development of new methods to identify genetic modifiers or interactions, and high-throughput functional assays in model systems.

## The complex genetics underlying normal-range human craniofacial morphology

Subtle variations characterize virtually every aspect of human facial morphology, the combinatorial possibilities resulting in a seemingly endless diversity of facial forms. The suggestion has been made that human faces are indeed more variable than the faces of other species and compared to other parts of our body [46]—a fact with potential evolutionary implications. It is therefore remarkable that so little is known about the biological factors underlying “normal-range” facial variation. There is no doubt that our genes play a major role; plain evidence for this can be found in the strong facial resemblance among members of our own families. More formally, we know from twin studies which aspects of our facial morphology are most and least heritable [47]. The genetic basis of human facial malformations and syndromes (discussed above) provides yet another line of evidence indicating the critical role of genes in shaping the human face.

The effort to identify the genes that influence normal-range facial phenotypes is important for several reasons. First, our understanding of how genes orchestrate facial morphogenesis is incomplete (discussed above). Gene mapping studies can provide developmental and cell biologists with new candidates for investigation (which genes to focus on). Moreover, such studies can reveal which aspects of facial morphology are likely to be impacted by specific variants and therefore the cell populations and tissues in which to look for effects. Second, an improved understanding of the genetic basis of normal-range variation can help elucidate the etiology of craniofacial malformations. Many craniofacial disorders are characterized by highly variable phenotypic expression. In Treacher Collins syndrome, for example, the same *TCOF1* mutation can impact the face with dramatically different levels of severity [48]. Such phenotypic variability may be due to the impact of many other normally functioning genes that influence how the face grows acting either independently or interactively with the mutated gene (and environmental factors) to produce a final outcome. Third, in the future, it may be possible to use information gleaned from facial gene mapping studies to create predictive models of facial features. We are not able to do this yet, but one can imagine the potential applications: recreating faces from DNA harvested from the bones of ancestors or left at a crime scene or residing in a genomic data repository like dbGaP, having the ability to predict an unborn child’s face or the face of a distant relative, or incorporating a patient’s genomic data into treatment planning at the orthodontist. Exploring such ideas is exciting but in many cases will be associated with important ethical and privacy implications.

The first genetic studies of normal-range human facial features were focused on candidate genes implicated in craniofacial syndromes or from transgenic mouse models with severe facial phenotypes [49–52]. Several of these early studies were featured in PLOS journals. The eventual emergence of large data sets containing both facial images—which allow facial morphology to be quantified in various ways—and dense genomic markers accelerated the pace of discovery by allowing for genome-scale analyses [53]. In 2012, the first GWAS of normal-range human facial shape were published [54–55]; one of these seminal studies by Liu and colleagues [55] appeared in *PLOS Genetics*. Of the handful of genome-wide significant loci identified by these 2 studies, the signal at *PAX3* was replicated. Common genetic variants at *PAX3* were associated with the shape of the nasal root—the uppermost part of the nose located between the eyes. This is noteworthy because mutations in *PAX3* can result in Waardenburg Syndrome Type 1, where one of the cardinal features is dysmorphology of the nasal root area. Several subsequent GWAS have now been conducted [56–59]. Each of these studies has identified a handful of loci, many of which contained at least one strong craniofacial candidate gene, associated with various measured facial traits. A recent study [60] using a novel data-driven approach to measuring 3D facial images based on machine learning principles identified 38 genome-wide significant loci, 15 of which were independently replicated. A number of the 15 replicated loci contained genes that have been implicated in early craniofacial development and in human craniofacial syndromes (e.g., *TBX15*, *SOX9*, *PAX3*, *DLX6*). An important finding from this study was that the effects of genetic variants on facial morphology tend to be highly specific, even within a single structural component like the nose. Another important finding was that variants at these 15 loci tended to show preferential activity in human cranial neural crest cells—a critical cell population for building the face.

As with most common traits [61], the implicated variants from the above facial GWAS tend to reside in noncoding (regulatory) regions of the genome. For example, many variants at the 15 aforementioned facial loci were in or near enhancers known to play a role in human craniofacial development [60]. This makes sense because variants that regulate the expression of intact genes—without altering the gene’s protein structure—would be expected to result in the kind of subtle modifications that characterize normal-range facial variation. This fact raises a number of challenges for identifying the likely functional variants, since much of the noncoding genome is still poorly characterized. Moreover, at any given locus, there may be many related variants that show evidence of association simply due to their proximity to the true functional variant(s). We expect this problem to only be exacerbated with the analysis of larger cohorts, which will likely reveal many more loci. The application of newer high-throughput functional approaches designed to probe the noncoding portions of the genome (CRISPR Cas9-based tools; STARR-seq) will be needed here to help sort through and make sense of these GWAS results [62].

Major breakthroughs are taking place in the area of craniofacial genetics, and we expect a rapid acceleration of discovery over the next few years. PLOS journals have played a key role in advancing the science, not only by providing a venue to publish cutting edge craniofacial research—some of which is highlighted in this collection—but also by making the availability of underlying data a key aspect and expectation of the publication process. The importance of open access and data sharing is critical for the transparency and reproducibility of our work and our field. These values help ensure that our scientific findings will eventually find their way to improving people’s lives.
